# Urinary concentration of selected nonpersistent endocrine disrupting chemicals—reproductive outcomes among women from a fertility clinic

**DOI:** 10.1007/s11356-023-25355-4

**Published:** 2023-01-26

**Authors:** Paweł Radwan, Bartosz Wielgomas, Michał Radwan, Rafał Krasiński, Stella Bujak-Pietrek, Kinga Polańska, Anna Kilanowicz, Joanna Jurewicz

**Affiliations:** 1 Department of Gynecology and Reproduction, “Gameta” Health Centre, 7 Cybernetyki St, 02-677 Warsaw, Poland; 2 Department of Gynecology and Reproduction, “Gameta” Clinic, Kielce-Regional Science –Technology Centre, 45 Podzamcze St, 26-060 Chęciny, Poland; 3 Department of Toxicology, Medical University of Gdańsk, 107 Hallera St., 80-416, Gdańsk, Poland; 4 Department of Gynecology and Reproduction, “Gameta” Hospital, 34/36 Rudzka St., 95-030 Rzgów, Poland; 5 Faculty of Health Sciences, Mazovian State University in Płock, 2 Dabrowskiego Sq., 09-402 Plock, Poland; 6 Department of Chemical Safety, Nofer Institute of Occupational Medicine, 8 Teresy St; 91-348, Łódź, Poland; 7 Department of Paediatrics and Allergy, Copernicus Memorial Hospital, Medical University of Lodz, Piłsudskiego 71; 90-329, Łódź, Poland; 8 Department of Toxicology, Medical University of Lodz, Muszynskiego 1; 90-151, Łódź, Poland

**Keywords:** Benzophenones, Exposure to endocrine-disrupting chemicals, Parabens, Reproductive outcomes

## Abstract

Parabens and benzophenones are compounds widely used in cosmetics and personal care products. Although human exposure is widespread there is a limited number of epidemiological studies assessing the relationship between exposure to these chemicals and female reproductive health. The aim of the study is to explore the relationship between paraben and benzophenone concentrations and reproductive outcomes among women attending a fertility center. This prospective cohort included 450 women undergoing in vitro treatment (IVF) at fertility clinic in Poland. The validated gas chromatography ion-tap mass spectrometry to assess concentrations of parabens in urine (methyl (MP), ethyl (EP), propyl (PP), butyl paraben (BP)) and benzophenone-3 (BP-3) was used. To explore the relationship between concentrations of examined chemicals and reproductive outcomes (methaphase II (MII) oocyte yield, total oocyte yield, implantation rate, fertilization rate, clinical pregnancy, live births), multivariable generalized linear mixed model was used for the analysis. Increased exposure to butyl paraben was associated with a significant decrease in MII oocyte count (*p* = 0.007) when exposure to BP was treated as the continuous variable. Additionally, the exposure to BP in the highest quartile of exposure also decreases MII oocyte count (*p* = 0.02) compared to the lowest quartile. Urinary concentrations of BP were not related to total oocyte count, fertilization and implantation rate, clinical pregnancy, and live birth when the exposure variable was continuous variable or in the quartiles of exposure. Exposure to MP, EP, PP, the sum of examined parabens, and benzophenone-3 were not related to any of the examined reproductive outcomes. Exposure to butyl paraben was associated with a decrease in MII oocyte count among women attending fertility clinic rinsing concerns that exposure may have a potential adverse impact on embryological outcomes. The results emphasize the importance to reduce chemicals in the environment in order to minimize exposure. As this is the first study showing such an association, further research is needed to confirm these novel results in other populations.

**Graphical Abstract:**

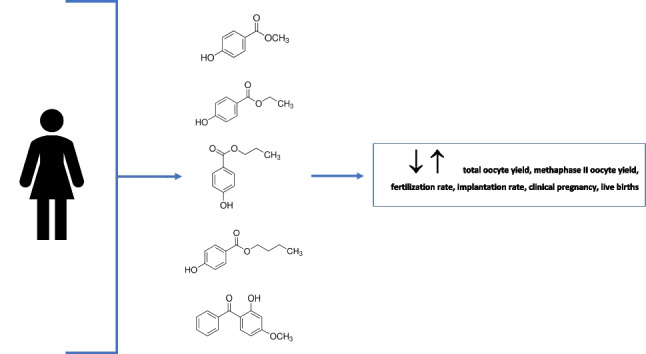

## Introduction

Widespread exposure to multiple natural and man-made chemicals which can act as endocrine-disrupting compounds (EDCs) raises concerns about the potential health effects of exposure, especially reproductive health. EDCs can interfere with endocrine functions, either through directly activating or inactivating endocrine target receptors or by disrupting the synthesis of hormones or the local control of active to inactive hormones by inhibiting or activating their metabolizing enzymes.

Because of the antifungal and antibacterial activities of parabens, they are commonly used in personal care products, food products, medicaments, and cosmetics (Giulivo et al. 2016; Andersen 2008). The antimicrobial and estrogenic activities of parabens depend on the alkyl substituent length (Giulivo et al. 2016). Human exposure to parabens occurs through different pathways such as ingestion, inhalation, and dermal absorption (Chen et al. 2007).

Parabens are considered endocrine disrupting compounds (EDCs), which have estrogenic activity and bind both estrogen receptors. Parabens are controlled in the EU United States, and Canada (Kolatorova et al. 2018). The maximum permissible level of parabens in cosmetic products is 0.4% for a single ester and 0.8% for a mixture of all parabens (Kolatorova et al. 2018).

Despite the fact that parabens have been found in the specimens taken from various groups of populations, the knowledge of their effect on human reproductive health is limited, especially in the female population. In animals treated orally with parabens, Vo et al. (2010) noticed histopathological changes in the ovaries and a decrease in ovarian weight and as well as altered estradiol and tetraiodothyronine (T4) in prepubertal female rats (Vo et al. 2010). On the other hand, paraben exposure was not associated with pregnancy outcome in mice (Shaw and deCatanzaro 2009) and rats (Boberg et al. 2008) and was not associated with the impact on weights of the reproductive organs and histopathological defects in female offspring (Kang et al. 2002).

A diminished ovarian reserve was observed in two studies after exposure to parabens (Smith et al. 2013; Jurewicz et al. 2020). Urinary concentrations of propyl paraben decrease antral follicle count and increase the FSH concentration (Smith et al. 2013; Jurewicz et al. 2020). Dodge et al. (2015) found a decrease in live birth after parabens exposure among women from fertility clinics, whereas Mínguez-Alarcón et al. (2016) found no relation between paraben exposure and in vitro fertilization outcomes (total and mature oocyte count, implantation rate, fertilization rate, clinical pregnancy, and live births).

Benzophenone-3 (BP-3), hydroxy-4-methoxybenzophenone, or oxybenzophenone, naturally occurring chemicals, are found in some flowering plants. BP-3 absorbs and scatters the ultraviolet (UV) rays which is why it is frequently used in sunscreens, lotions, cosmetics, indirect food additives, and fragrance enhancers. BP-3 is defined as an endocrine-disrupting chemical because of its similarities to the steroid hormone receptors (Schreurs et al. 2002). In UE, an acceptable dose of BP-3 is 6% in UV filters, and in the USA, Japan, and South Korea 5% (Balazs et al. 2016). Although BP-3 is widely used, studies on the adverse health effects of exposure in both animals and humans are scarce. Animal studies demonstrated that exposure to BP-3 decreased the number of oocytes, nests per ovary, and early primary follicles (Santamaría et al. 2019); lengthened the estrous cycles and decreased epididymal sperm density (French 1992); and disrupted the sperm development in the testicles of male offspring (Nakamura et al. 2015). Only in one study the statistically significant association between urinary levels of BP-3 and reproductive health in humans was noticed. Kunisue et al. (2012) observed that exposure to BP-3 was related to endometriosis, whereas no association was observed in case of infertility (Chen et al. 2013), time to pregnancy (Buck Louis et al. 2014), or secondary sex ratio (Bae et al. 2016). On the other hand, a higher rate of clinical pregnancy, implantation, and live births was observed in relation to BP-3 exposure among subjects from fertility clinics (Mínguez-Alarcóna et al. 2019).

Give the ubiquitous exposure to parabens and benzophenone-3 among women of reproductive age the potential effect of exposure on parameters of women’s fecundity such as markers of ovarian reserve, hormonal stimulation, fertilization and implantation rate, clinical pregnancy, and live birth remain unclear. Studies on exposure to parabens and benzophenone-3 are scarce, and their results are inconsistent and inconclusive; thus, the study aims to evaluate the relationship between urinary concentrations of parabens and benzophenone and reproductive outcomes among women from fertility centers. To the best of our knowledge, this is the first study to examine the association between concentrations of different parabens (methyl, ethyl, propyl, butyl) and benzophenone-3 in urine and IVF outcomes in one study. Additionally, the exposure to ethyl parabens and early reproductive outcomes have never been assessed.

## Materials and methods

### Study population

This prospective cohort examines environmental exposure to widespread endocrine-disrupting chemicals and fertility among couples undergoing fertility treatments (Radwan et al. 2021). The study included 450 women with 674 IVF cycles at the Assisted Reproduction Center of Lodz, Poland, from January 2017 to December 2019. The inclusion criteria were body mass index (BMI) of less than 40 kg/m^2^, ages ≥ 45 years old, and at least one finished IVF treatment cycles. The exclusion criteria were thyroid dysfunction (TSH > 2.5 µU/mL) and chlamydia infection as previously described Radwan et al. (2020). We follow prospectively women during their IVF treatment. Nofer Institute of Occupational Medicine, Lodz, Poland, Bioethical Committee approved the study protocol, and study subjects received informed consent before participation (resolution no 23/2016).

Information about height and weight was collected with body mass index (BMI) calculation. Study women were asked to fill out a questionnaire about demographic characteristics, lifestyle, environmental and occupational factors, and medical data.

### ART cycle procedures—embryological outcomes and reproductive hormone assessment

All the study participants were assigned standard fertility diagnosis by a physician codded in agreement with the definitions of the Society for Assisted Reproductive Technology. All the medical and reproductive information was abstracted from the patient’s medical electronic cards.

Chemiluminescence immunoassay was used to measure the concentrations of estradiol (E2), progesterone, luteinizing hormone (LH), and follicle-stimulating hormone (FSH) hormones in serum, collected between the second and third days of the menstrual cycle.

Depending on the female BMI, ovarian reserve and age-specific protocol was used for ovulation stimulation (long agonist or short antagonist) as previously described (Radwan et al. 2020). Embryologists classified the total number of oocytes retrieved per cycle as degenerated, germinal vesicle (GV), methaphase I (MI), and methaphase II (MII). After 17–20 h of insemination fertilization rate was assessed by the embryologists as the number of oocytes with two pronuclei divided by the number of MII oocytes. The percentage of embryos with successful implantation compared to the number of embryos transferred was defined as the implantation rate. Clinical pregnancy was related to an elevation of β-hCG with confirmation of an intrauterine pregnancy on an ultrasound at 6 weeks. The birth of a neonate on or after 24 weeks of gestation was defined as live birth.

### Urine collection and parabens and benzophenone-3 exposure assessment

Women provided one (32%) or two (68%) spot urine samples per IVF cycle at the beginning and in the middle of the IVF cycle in a polypropylene specimen cup. Cycle-specific urinary concentration was assessed as the geometric mean of the parabens and benzophenone-3 concentrations from two spot urine samples except for cycles with only one urine sample.

The calibrated handheld refractometer was used to measure the specific gravity (SG) after measuring SG the urine samples were frozen at − 20 °C and sent to the laboratory. Parabens (methyl, ethyl, propyl, butyl) and benzophenone-3 were analyzed by GC–MS/MS method with LOD 0.1 µg/L for each chemical as presented elsewhere (Jurewicz et al. 2020). The standard stock solutions (1 mg/ml) of each paraben and benzophenone-3 were prepared in acetonitrile. The stock solutions were used to prepare two separate working solutions: one for the fortification of quality control urine samples and the other one for the calibration. All solutions were stored at − 20 °C in the dark and were stable for at least 6 months described by Lu et al. (2015) with some modifications. The method allows to evaluate the total concentration (free plus conjugated) of analyzed parabens in urine samples. Since, the major source of parabens to humans are cosmetic products, personnel handling urine samples was instructed to avoid paraben-containing personal care products during the sample collection, sample preparation, and analysis to minimize the risk of external contamination. A validated gas chromatography ion-tap mass spectrometry was used (Varian GC-450) equipped with low bleed VF5-ms capillary column (30 m × 0.25 mm × 0.25 μm + 10 m EZ-guard, Varian) and 1177 split/splitless injector (isothermal condition: 280 °C). The oven column program was 60 °C (3 min), 60 °C–140 °C (120 °C/min), 140 °C–290 °C (17 °C/min), and 280 °C (13 min). Tandem mass spectrometry (ion trap mass spectrometer, Varian 220-MS) was applied as a detection method as described elsewhere (Jurewicz et al. 2020). Below the limit of detection (LOD), sample concentrations were replaced with LOD divided by the square root of 2 (Hornung and Reed 1990).

### Statistical analysis

Characteristics of the patients and reproductive outcomes of the cycles are described using mean with standard deviation (SD) or counts (%). Urinary concentrations of parabens (methyl, ethyl, propyl, butyl) and benzophenone-3 were categorized as follows: < LOD to 25th (reference category), > 25th to 50th, > 50th to 75th, > 75th, and as a continuous variable. Specific gravity (SG) was used to adjust for urine dilution. The correlation between urinary concentrations of chemicals was assessed using Spearman statistics.

Generalized estimating equation (GEE) models were used to analyze the relationship between exposure to environmental chemicals and with pregnancy outcomes (including total oocyte yield, MII oocyte count, implantation rate, fertilization rate, clinical pregnancy, live birth rate) taking into account more than one cycle from the same patients and correlation between repeated embryo transfer cycles. The generalized estimating equation models are mostly used in longitudinal studies where we have repeated measurements through time, whereas cross-sectional studies are a single outcome per individual. Observations from an individual tend to be correlated and the correlation must be taken into account for valid inference. We account for correlation in outcomes across multiple IVF cycles per woman and adjust for potential confounders.

Variables such as female age, BMI, SG, infertility diagnosis, smoking, antral follicle count (AFC), and protocol type were treated as the confounding variables and included in the model.

The R statistical software (version 3) was used for statistical assessment with a two-sided 5% level of significance (R Core Team 2016).

## Results

### Participants’ characteristics and reproductive outcomes

A total of 450 women undergoing 674 cycles were included in this study. Table 1 summarizes the detailed characteristics of the study population. The mean age of women was 31.28 years old, and the average BMI was 23.19 kg/m^2^. Most women (65.11%) had higher education, were not smokers (92%), and drank none or less than 1 drink/week (54.44%). Duration of a couple’s infertility least mostly more than 5 years (36.44%). In most cases, the initial infertility diagnosis was the male factor (38%). GnRH-antagonist protocol was used for stimulation of ovulation (55.90%). The mean antral follicle count in both ovaries was 12.54 ± 7.21. The mean level of examined hormones: FSH, E2 peak, E2, LH, progesterone, and AMH, was 6.21 ± 1.13 IU/l, 2608.74 ng/ml, 92.78 ± 15.78 pg/ml, 5.34 ± 3.24 IU/l, 0.99 ± 1.58 ng/ml, and 1.19 ng/ml, respectively (Table 1).

**Table 1:** Baseline characteristics and IVF outcome among the study population *N* = 450

**Variables**	
Per women	
Education (*n* (%))	
Vocational	13 (2.89)
Secondary	144 (32.00)
Higher	293 (65.11)
Age [years] (*n* (%)) 24–30	81 (18.00)
31–39	342 (76.00)
40–44	27 (6.00)
Mean ± SD	31.28 ± 3.52
BMI [kg/m^2^] (*n* (%))	
< 18.5	26 (5.78)
18.5–24.9	261 (58.00)
25–29.9	135 (30.00)
30–40	28 (6.22)
Mean ± SD	23.19 ± 2.67
Current smoking (*n* (%))	
No	414 (92.00)
Yes	36 (8.00)
Alcohol use	
None or < 1 drink/week	245 (54.44)
1–3 drinks/week	198 (44.0)
Everyday	7 (1.56)
Duration of couple’s infertility [years] (*n* (%))	
1–2	34 (7.56)
2–3	121 (26.89)
3–5	131 (29.11)
> 5	164 (36.44)
Per cycle	
Initial infertility diagnosis (*n* (%))	
Male factor	171 (38.0)
Idiopathic	139 (30.89)
Endometriosis	62 (13.78)
Ovarian factor	21 (4.67)
Tubal factor	46 (10.22)
Missing data	11 (2.44)
Stimulation protocol	
Long GnRH agonist protocol	297 (44.10%)
GnRH-antagonist protocol	377 (55.90%)
AFC (*n*) (mean ± SD)	12.54 ± 7.21
FSH (IU/l) (mean ± SD)	6.21 ± 1.13
E2 peak (ng/ml) (mean ± SD)	2608.74 ± 1614.63
E2 (pg/ml) (mean ± SD)	92.78 ± 15.78
Progesterone (ng/ml) (mean ± SD)	0.99 ± 1.58
LH (IU/l) (mean ± SD)	5.34 ± 3.24
AMH (ng/ml) (mean ± SD)	1.19 ± 1.22
Number of oocyte yield (mean ± SD)	13.21 ± 7.44
Number of MII oocytes (mean ± SD)	9.98 ± 7.22
Fertilization rate (%) (mean ± SD)	80 ± 21
Implantation rate (%) per embryo (mean ± SD)	38.16 ± 2.79
Number of top-quality embryos (mean ± SD)	2.01 ± 2.48
Number of embryos transferred (mean ± SD)	1.95 ± 0.16
Clinical pregnancy rate (%) per cycle (mean ± SD)	43.12 ± 7.89

*SD* standard deviation, *AMH* Anti-Müllerian hormone, *AFC* antral follicle count, *FSH* follicle-stimulating hormone, *E2* estradiol, *LH Luteinizing hormone*

The characteristics of IVF outcomes are presented in Table 1. The mean ± SD number of total oocyte yield, MII oocytes, fertilization rate, number of top quality embryos, number of embryo transferred, implantation rate, clinical pregnancy was 13.21 ± 7.44, 9.98 ± 7.22, 80 ± 21, 2.01 ± 2.48, 1.95 ± 0.16, 38.16 ± 2.79, and 43.12 ± 7.89, respectively (Table 1).

### Environmental parabens and benzophenone-3 exposure

We collected 739 urine samples from 450 participants and from 85% of women (*N* = 573) two urine samples were collected. The distribution of examined non-persistent endocrine-disrupting chemicals is presented in Table 2. The detection frequency was the highest in the case of MP (94.22%), followed by BP-3 (94.12%), EP (89.15%), PP (89.07%), and BP (67.12%) (Table 2). The geometric mean urinary concentration was the highest in the case of MP (83.22 ± 5.87 ng/ml) than EP (10.32 ± 5.12 ng/ml), PP (9.22 ± 2.13 ng/ml), BP-B (6.89 ± 8.73 ng/ml), and BP (3.34 ± 2.55 ng/ml) (Table 2). The correlation between all examined chemicals was statistically significant, except for EP and BP-3 (*p* = 0.05) (Table 3).

**Table 2:** Distribution of urinary concentration of non-persistent endocrine disrupting chemicals among 450 women contributing 739 urine samples

Metabolites of chemicals in urine	Statistics							
	A mean (SD)	G mean (GSD)	LOD	Q25	Median	Q75	Q95	Detection frequency (%)
ng/ml								
MP	123.54 (245.81)	83.22 (5.87)	0.1	23.16	90.88	146.77	422.11	94.22
EP	58.22 (71.05)	10.32 (5.12)	0.1	1.00	8.77	57.22	234.98	89.15
PP	48.25 (72.18)	9.22 (2.13)	0.1	2.09	8.54	30.21	180.76	89.07
BP	9.34 (20.15)	3.34 (2.55)	0.1	1.22	3.12	6.20	39.11	67.12
BP-3	86.09 (306.42)	6.89 (8.73)	0.1	1.47	4.96	24.11	481.27	94.12
SG adjusted (ng/ml)								
MP	147.25 (221.17)	95.14 (3.21)	0.1	28.17	96.11	198.56	534.11	94.22
EP	61.13 (156.18)	11.36 (6.10)	0.1	1.98	8.97	59.18	255.89	89.15
PP	49.11 (81.10)	9.78 (2.10)	0.1	2.17	8.78	37.12	189.78	89.07
BP	9.78 (20.23)	3.67 (1.98)	0.1	1.78	4.12	7.76	43.78	67.12
BP-3	92.48 (288.85)	8.83 (7.80)	0.1	1.99	2.12	6.14	28.29	94.12

Per IVF cycle (*N* total = 674), 1 (*N* = 101; 15%), or 2 (*N* = 573; 85%)

*A Mean* artimetic mean, *G Mean* geometric mean, *Q25* 25 quartile, *Q75* 75 quartile, *Q95* 95 quartile, *LOD* limit of detection, *MP* methyl paraben, *EP* ethyl paraben, *PP* propyl paraben, *BP* butyl paraben, *BP-3* benzophenone-3

**Table 3:** Spearman correlation between urinary concentration of non-persistent endocrine disrupting chemicals

	MP	EP	PP	BP	BP-3
MP	1	** < 0.001**	** < 0.001**	** < 0.001**	** < 0.001**
EP	0.55	1	** < 0.001**	** < 0.001**	0.055
PP	0.64	0.41	1	** < 0.001**	** < 0.001**
BP	0.30	0.28	0.28	1	** < 0.001**
BP-3	0.20	0.09	0.17	0.15	1

Above diagonal—p values; below diagonal—correlations

*EP* ethyl paraben, *PP* propyl paraben, *BP* butyl paraben, *iBuP* izobuthyl paraben, *BP-3* benzophenone-3

Bold values-significance level 0.05 was used for statistical interference

### Urinary concentration of selected chemicals in relation to reproductive outcomes

Table 4 presents the relationships of exposure to selected chemicals and IVF outcomes. Increased exposure to butyl paraben was associated with a significant decrease in MII oocyte count (*p* = 0.007) when the exposure to BP was treated as the continuous variable. Additionally, the exposure to BP in the highest (> 75 percentile) quartile of exposure also decrease MII oocyte count (*p* = 0.02) compared to the lowest (≤ 25 percentile) quartile. Urinary concentrations of BP were not related to total oocyte count, fertilization and implantation rate, clinical pregnancy, and live birth when the exposure variable was continuous variable or in the quartile of exposure. Exposure to MP, EP, and PP and the sum of examined parabens and benzophenone-3 were not related to IVF outcomes, specially total oocyte yield and MII oocyte count. Additionally, urinary paraben concentrations were not significantly related to the rates of implantation, clinical pregnancy, fertilization, and live births (Table 4).

**Table 4:** Urinary concentrations of selected chemicals in relation to IVF outcomes among 450 women undergoing 674 cycle

	Cat	Total oocyte yield, coef (95%CI); *p*	MII oocyte count, coef (95%CI); *p*	Fertilization rate, coef (95%CI); *p*	Implantation rate, coef (95%CI); *p*	Clinical pregnancy, coef (95%CI); *p*	Live birth, coef (95%CI); *p*
MP	Cont	− 0.02 (− 0.16; 0.13); 0.82	0.01 (− 0.14; 0.17); 0.86	0.08 (− 0.07; 0.23); 0.28	− 0.02 (− 0.17; 012); 0.74	− 0.05 (− 0.18; 0.09)0.51	− 0.07 (− 0.22; 0.08); 0.34
	Q2	− 0.04 (− 0.21; 0.12); 0.61	0.11 (− 0.04; 0.25); 0.15	0.10 (− 0.06; 0.26); 0.20	0.10 (− 0.05; 0.26); 0.18	0.06 (− 0.08; 0.19); 0.41	− 0.06 (− 0.21; 0.10); 0.47
	Q3	0.01 (− 0.14; 0.17); 0.85	− 0.04 (− 0.17; 0.1); 0.61	0.08 (− 0.06; 0.22); 0.26	− 0.03 (− 0.20; 0.13); 0.70	0.09 (− 0.06; 0.23); 0.24	− 0.08 (− 0.21; 0.058); 0.26
	Q4	− 0.04 (− 0.18; 0.11); 0.64	0.03 (− 0.12; 0.18); 0.67	− 0.02 (− 0.16; 0.11); 0.74	− 0.02 (− 0.192; 0.16); 0.86	− 0.08 (− 0.23; 0.06); 0.24	− 0.08 (− 0.22; 0.06); 0.28
EP	Cont	− 0.07 (− 0.22; 0.076); 0.34	− 0.06 (− 0.20; 0.07); 0.36	− 0.04 (− 0.19; 0.10); 0.56	− 0.08 (− 0.27; 0.11); 0.41	− 0.15 (− 0.33 − 0.03); 0.10	− 0.11 (− 0.28; 0.07); 0.22
	Q2	− 0.06 (− 0.21; 0.10); 0.47	− 0.04 (− 0.180; 0.10); 0.55	0.04 (− 0.09; 0.18); 0.52	− 0.01 (− 0.20; 0.18); 0.90	0.02 (− 0.16; 0.20); 0.82	0.10 (− 0.07; 0.28); 0.24
	Q3	− 0.08 (− 0.21; 0.06); 0.26	− 0.06 (− 0.20; 0.08); 0.37	0.04 (− 0.10; 0.17); 0.60	− 0.01 (− 0.16; 0.14); 0.93	0.06 (− 0.14; 0.26); 0.54	0.04 (− 0.13; 0.21); 0.64
	Q4	0.02 (− 0.13; 0.18); 0.79	− 0.001 (− 0.14; 0.14); 0.98	0.08 (− 0.06; 0.22); 0.26	0.12 (− 0.07; 0.31); 0.22	0.04 (− 0.13; 0.22); 0.62	− 0.02 (− 0.20; 0.16); 0.84
PP	Cont	− 0.16 (− 0.35; 0.034); 0.12 −	0.03 (− 0.16; 0.21); 0.77	− 0.06 (− 0.23; 0.11); 0.47	0.02 (− 0.15; 0.18); 0.84	− 0.07 (− 0.34; 0.20); 0.62	0.06 (− 0.13; 0.25); 0.53
	Q2	0.10 (− 0.04; 0.23); 0.17	0.07 (− 0.09; 0.24); 0.39	− 0.05 (− 0.23; 0.14); 0.62	− 0.06 (− 0.23; 0.11); 0.51	0.06 (− 0.12; 0.25); 0.50	0.09 (− 0.10; 0.29); 0.33
	Q3	− 0.03 (− 0.19; 0.12); 0.68	0.04 (− 0.13; 0.21); 0.65	− 0.16 (− 0.35; 0.03); 0.01	− 0.06 (− 0.24; 0.12); 0.52	− 0.23 (− 0.47; 0.01); 0.07	− 0.05 (− 0.25; 0.16); 0.67
	Q4	0.65 (− 0.08; − 0.23); 0.08	0.01 (− 0.17; 0.18); 0.96	− 0.06 (− 0.23; 0.11); 0.50	− 0.21 (− 0.37; − 0.04); 0.02	0.010 (− 0.14; 0.33); 0.42	− 0.02 (− 0.20; 0.173); 0.87
BP	Cont	− 0.01 (− 0.22; 0.20); 0.91	− 0.20 (− 0.34; − 0.05); **0.007**	− 0.05 (− 0.24; 0.134); 0.57	0.04 (− 0.07; 0.15); 0.49	0.04 (− 0.11; 0.19); 0.57	0.07 (− 0.08; 0.22); 0.36
	Q2	0.01 (− 0.20; 0.22); 0.93	0.10 (− 0.10; 0.30); 0.32	− 0.03 (− 0.17; 0.12); 0.73	0.09 (− 0.08; 0.26); 0.29	0.06 (− 0.09; 0.21); 0.47	0.07 (− 0.08:0.21); 0.39
	Q3	0.15 (− 0.07; 0.36); 0.19	− 0.13 (− 0.32; 0.06); 0.16	− 0.07 (− 0.21; 0.082); 0.38	0.08 (− 0.06; 0.21); 0.27	0.05 (− 0.1; 0.21); 0.50	− 0.05 (− 0.20; 0.10); 0.53
	Q4	0.11 (− 0.08; 0.29); 0.26	− 0.21 (− 0.39; − 0.03); **0.02**	− 0.09 (− 0.24; 0.07); 0.26	0.003 (− 0.12; 0.11); 0.96	− 0.04 (− 0.19; 0.12); 0.57	0.08 (− 0.08; 0.23); 0.33
BP3	Cont	− 0.04 (− 0.19; 0.11); 0.58	− 0.10 (− 0.25; 0.06); 0.22	− 0.02 (− 0.17; 0.14); 0.81	− 0.02 (− 0.18; 0.13); 0.78	0.01 (− 0.14; 0.17); 0.87	− 0.05 (− 0.20; 0.09); 0.51
	Q2	0.003 (− 0.15; 0.16); 0.97	0.04 (− 0.11; 0.19); 0.59	0.03 (− 0.13; 0.18); 0.75	− 0.04 (− 0.19; 0.12); 0.65	− 0.12 (− 0.28; 0.03); 0.12	− 0.02 (− 0.17; 0.13); 0.77
	Q3	0.32 (− 0.02; − 0.17); 0.13	− 0.09 (− 0.24; 0.06); 0.25	0.05 (− 0.09; 0.19); 0.51	0.05 (− 0.09; 0.2); 0.51	− 0.03 (− 0.18; 0.12); 0.69	0.03 (− 0.13; 0.18); 0.73
	Q4	− 0.18 (− 0.32; 0.04); 0.56	− 0.04 (− 0.28; 0.194); 0.72	− 0.03 (− 0.27; 0.21); 0.84	− 0.07 (− 0.32; 0.17); 0.53	0.12 (− 0.11; 0.36); 0.30	0.16 (− 0.06; 0.40); 0.16
Sum of parabens	Cont	0.35 (− 0.03; − 0.18); 0.12	− 0.04 (− 0.28; 0.19); 0.72	0.17 (− 0.07; 0.40); 0.16	0.12 (− 0.11; 0.36); 0.31	− 0.08 (− 0.31; 0.16); 0.53	− 0.02 (− 0.26; 0.216); 0.84
	Q2	0.06 (− 0.08; 0.22); 0.65	− 0.04 (− 0.23; 0.23); 0.97	− 0.02 (− 0.25; 0.213); 0.85	0.02 (− 0.23; 0.24); 0.98	0.09 (− 0.23; 0.25); 0.94	0.02 (− 0.21; 0.26); 0.83
	Q3	0.46 (− 0.07; − 0.228); 0.08	− 0.16 (− 0.40; 0.074); 0.17	0.14 (− 0.09; 0.38); 0.23	0.02 (− 0.21; 0.26); 0.83	− 0.18 (− .41; 0.055); 0.133	0.01 (− 0.21; 0.25); 0.87
	Q4	0.36 (0.05 − 0.09); 0.20	− 0.05 (− 0.29; 0.17); 0.62	0.01 (− 0.22; 0.24); 0.92	0.06 (− 0.17; 0.30); 0.57	0.10 (− 0.12; 0.34); 0.37	− 0.12 (− 0.36; 0.11); 0.29

*EP* ethyl paraben, *PP* propyl paraben, *BP* butyl paraben, *iBuP* izobuthyl paraben, *BP-3* benzophenone-3, *con* continuous variable

Q2 (25–50] percentile; Q3 (50–75] percentile, Q4 > 75 percentile; reference category to Q2, Q3, snfQ4 is Q1 Q1 ≤ 25 percentile; model adjusted for SG, BMI, age, smoking, infertility diagnosis, protocol type, and AFC

Reference groups: 1, in case > LOD, reference is < LOD; 2, in case Q2, Q3, and Q4, reference is Q1

Bold values-significance level 0.05 was used for statistical interference

## Discussion

This study was designed to analyze the relationship between exposure to parabens and benzophenoe-3 with IVF outcomes (total oocyte yield, MII oocytes, implantation, fertilization, rate, clinical pregnancy, and live births). It was demonstrated that increased urinary concentrations of BP were significantly related to MII oocyte count. To the best of our knowledge, this is the first study to examine the association between concentrations of parabens and benzophenone-3 in urine and IVF outcomes in one study. Additionally, the exposure to ethyl parabens and early reproductive outcomes have never been assessed.

Although exposure to parabens and benzophenones is widespread, a surprisingly small number of epidemiological studies evaluate the potential effect of exposure on female reproductive health, especially early embryological or reproductive outcomes. The exposure to parabens and reproductive outcomes was evaluated in three studies (Dodge et al., 2015; Sabatini et al. 2011; Mínguez-Alarcón et al. 2016; Dodge et al. 2015). Additionally, one study explored the mixture exposure where one of the compounds is parabens (Mínguez-Alarcóna et al. 2019). Total oocyte yield, MII oocyte, endometrial thickness, fertilization rate, embryo quality, implantation, clinical pregnancy, and live births were evaluated in these studies (Dodge et al. 2015; Mínguez-Alarcón et al. 2016; Mínguez-Alarcóna et al. 2019).

In the study performed by Dodge et al. (2015), it was observed that paternal concentrations of methyl paraben in the second quartile were associated with decreased odds of live birth following IUI (intrauterine insemination) (OR = 0.19; 95% CI: 0.04, 0.82). On the other hand, Mínguez-Alarcón et al. 2016 found no association between levels of methyl paraben, propylparaben and butylparaben in urine and IVF outcomes (proportion of high embryo, quality, total and mature oocyte counts, clinical pregnancy, fertilization, implantation rate, and live births). In the study performed by the same author in 2019 when the mixture exposure was studied (phthalates, bisphenol A, and parabens), there was no association between parabens and any examined IVF outcomes (Mínguez-Alarcóna et al. 2019). The differences in results of our findings with the studies may be associated with a bigger sample size (245 women and 356 IVF cycles versus 450 women and 674 cycles in the present study), higher exposure level in case of BP (median: 1.18 ng/ml versus 3.12 in our study) and some different confounding factors used for the statistical analysis (using additional confounding such as protocol type and antral follicle count). In the present study, we observed higher urinary concentrations of parabens: MP, EP, and BP compared to US women of the same age from the general population in the fourth report of the National Health and Nutrition Examination Survey (NHANES) (CDC 2018). The median concentrations for MP, EP, and BP were 90.88 μg/L in our study and 73.9 μg/L in NHANES, 8.77 μg/L and 1.60 μg/L and 3.12 μg/L and < LOD, respectively. PP geometric mean was lower in the present study 9.22 μg/L and 13.5 μg/L in NHANES. Additionally, the examined endocrine-disrupting chemicals have been assessed in 53 pregnant women in the Indian population. The concentrations of methyl paraben (MP; 20.92 ng/mL and 18.92 ng/mL), ethyl paraben (EP; 1.97 ng/mL and 1.89 ng/mL), propyl paraben (PP; 19.22 ng/mL and 18.82 ng/mL), butyl paraben (BP; 1.11 ng/mL and 1.37 ng/mL) in maternal blood and amniotic fluid which suggest that maternal exposure is positively associated with in utero exposure to the developing fetus (Shekhar et al. 2017). A decrease in live births was observed after the administration of BP to pregnant rats (Kang et al. 2002). Additionally, weight change in the ovaries of Sprague–Dawley rats was observed (Daston 2004). In the study performed by Vo et al. (2010) in rats exposed to paraben abnormalities in uterus morphology and in ovarian histopathology, decreased length of the estrous cycle and serum estradiol levels was noticed. MP altered porcine oocyte morphology and caused a reduction in cumulus cell expression which resulted in decreased oocyte maturation (Barajas-Salinas et al. 2020). On the other hand, no effect of BP exposure was observed in case of fecundity, fertility, and reproductive parameters in different generations of rats (F0, F1, or F2) (Hubbard et al. 2000). Additionally, no relation of BP and PP exposure with implantation sites and offspring survival was observed (Shaw and deCatanzaro 2009). In rats, BP was not related to maternal ovarian E2 level (Shaw and deCatanzaro 2009).

Only one epidemiological evaluated the association between benzophenone-3 and IVF treatment among women from fertility clinic (Mínguez-Alarcóna et al. 2019). A higher rate of clinical pregnancy, implantation, and live births was associated with BP-3 exposure. This relation may be due to the factors not considered in the analysis related to spending time outdoors which may have a positive effect on reproduction rather than the beneficial effect of BP-3. In the present study, no association was found between BP-3 and examined reproductive outcomes. No association may be due to very low exposure to BP-3 in the Polish population. The median urinary concentration is 4.96 ng/ml compared to 58.46 ng/ml in the study by Mínguez-Alarcóna et al. (2019). In general, the concentration of BP-3 is low in the present study compared to the urinary concentration among Porto Rican women (Meeker et al. 2013) or in National Health and Nutrition Examination Survey (NHANES) study among US females general population in 2013–2014 (CDC 2018) (median: 31.3, 29.7 ng/ml respectively).

Other epidemiological studies found no association, between exposure to BP-3 and time to pregnancy among couples who were discontinuing the use of contraceptives in order to become pregnant (Buck Louis et al. 2014). In this study, only male BP-2 concentration reduced fecundity (Buck Louis et al. 2014). In the same cohort, the exposure to type-BP filers and the secondary sex ratio was evaluated (Bae et al. 2016), and similarly no association was found for BP-3 urinary concentration but not for BP-2 and BP-4. Contrary to this finding, animal studies indicated that exposure to BP-3 decreased the number of oocytes, nests per ovary, and early primary follicles, leading to perturbation in the early germ cell development (Santamaría et al. 2019). In other studies, BP-3 caused lengthened estrous cycles and decreased epididymal sperm density in rodents (French 1992).

Given the ubiquitous exposure to parabens and benzophenone-3 among women of reproductive age the potential effect of exposure on parameters of women’s fecundity such as markers of ovarian reserve, hormonal stimulation, fertilization and implantation rate, clinical pregnancy, and live birth remain unclear. The studies on exposure to parabens and benzophenone-3 are scarce, and their results are inconsistent and inconclusive.

The current study has some limitations. Exposure misclassification is possible given the short biological half-lives of these non-persistent chemicals. However, two urine samples were collected for the vast majority of the participants, and using the average measurement of the two samples will minimize the effect of high within-person variability and reduce exposure misclassification. It is difficult to generalize the study results, but the findings can be applicable to women with IVF treatment. As this is the epidemiological study design (prospective cohort), we were not able to find the mechanism of association between exposure and outcomes. The study have also some strengths associated with the prospective study design, using the standardized protocol and detailed questionnaire which allowed to control the confounding factors. Additionally, the study was performed in one center, which help the recruitment, and data collection was performed in the same way to allow comparisons.

In conclusion, urinary concentrations of butyl paraben were related to a decrease in MII oocyte count among women from fertility clinic rinsing concern that exposure may have a potential adverse impact on embryological outcomes. The results emphasize the importance to reduce chemicals in the environment in order to minimize the exposure. As this is the first study showing such an association, further research is needed to confirm these novel results in other populations and larger and more diverse cohorts. It could be important to investigate the exposure to mixtures of environmental chemicals, within both individuals and couples would further elucidate the early reproductive outcomes of these chemicals. Mechanistic studies are also needed to better understand potential health effects.

## Data Availability

The datasets used and analyzed during the current study are available from the corresponding author upon reasonable request.
